# Perspectives on strengthening local food systems in Small Island Developing States

**DOI:** 10.1007/s12571-022-01281-0

**Published:** 2022-05-04

**Authors:** Cornelia Guell, Catherine R. Brown, Otto W. Navunicagi, Viliamu Iese, Neela Badrie, Morgan Wairiu, Arlette Saint Ville, Nigel Unwin, the Community Food and Health (CFaH) team, Sashi Kiran, T. Alafia Samuels, Ian Hambleton, Colin Tukuitonga, Connie Donato-Hunt, Florian Kroll, Rachel Nugent, Nita G. Forouhi, Sara Benjamin-Neelon

**Affiliations:** 1 University of Exeter, European Centre for Environment & Human Health, Truro, UK; 2 The University of the West Indies, George Alleyne Chronic Disease Research Centre, Bridgetown, Barbados; 3 Pacific Centre for Environment and Sustainable Development, The University of the South Pacific, Suva, Fiji; 4 Department of Food Production, The University of the West Indies, St. Augustine, Trinidad and Tobago; 5 Department of Geography, The University of the West Indies, St. Augustine, Trinidad and Tobago; 6 Medical Research Council Epidemiology Unit, University of Cambridge, Cambridge, UK

**Keywords:** Small-scale food production, Food systems, Food sovereignty, Nutritious diets, Caribbean, Pacific

## Abstract

Small Island Developing States (SIDS) share high burdens of nutrition-related conditions, including non-communicable diseases, associated with an increasing reliance on imported, processed foods. Improving health through increasing the production and consumption of local, nutritious foods is a policy objective of many SIDS governments. This study aimed to understand contemporary challenges and opportunities to strengthening local food systems in two case study settings, Fiji and St. Vincent and the Grenadines. Fifty-two in-depth, semi-structured interviews were conducted with key stakeholders involved in local food production. Interviews were analysed by both country teams using thematic analysis. Local food production networks in both settings included formal governance bodies as well as more informal connections through civil society and communities. Their main function was the sharing of resources and knowledge, but levels of trust and cooperation between the stakeholders varied in a market open to intense competition from imports. Local food production was hindered by few and slow investments by local governments, dated technology, and lack of knowledge. Stakeholders believed this marginalisation was occurring against a background of rising preferences for imported foods in the population, and increasing disinterest in employment in the sector. Despite the challenges, strong narratives of resilience and opportunity were highlighted such as national pride in local produce for commercialisation and local diets. Efforts to support local food production in SIDS should focus on strengthening governance structures to prioritise local produce over corporate and import markets, assist collaboration and co-learning, and support alternative agro-food practices.

**Supplementary Information:**

The online version contains supplementary material available at 10.1007/s12571-022-01281-0.

## Introduction

Small Island Developing States (SIDS) are a group of 58 countries facing specific social, economic and environmental vulnerabilities including complex food and nutrition-related challenges. SIDS were recognised as a distinct group at the 1992 United Nations Conference on Environment and Development. While overnutrition increasingly displaces undernutrition in most SIDS, access to quality food remains a critical issue for food security, population health and social and economic development (FAO, 2016b). SIDS have experienced a ‘nutrition transition’ over the past several decades; locally grown traditional foods have been replaced in the diet by imported, predominantly calorie-dense, processed and ultra-processed foods (Tu'akoi et al., 2018). As a result, obesity-related non-communicable diseases (NCDs) such as diabetes mellitus and cardiovascular diseases have become a serious burden in SIDS (FAO et al., 2017). For example, 33% of the Caribbean population are classified as obese, and nearly half of Pacific SIDS report an age-adjusted diabetes prevalence of > 20% (FAO et al., 2017; International Diabetes Federation, 2019). While there are other important risk factors for NCDs, including smoking, physical inactivity and excess alcohol consumption, aspects of diet and their sequelae are the major contributors to NCD risk in the Caribbean and Pacific (Global Burden of Disease Collaborative Network, 2021).

SIDS rely largely on food imports over local food production, with over 60% of food imported in Caribbean and Pacific countries, and over half of these countries importing over 80% of food (FAO et al., 2017). Top foods imported are processed foods, followed by wheat, corn, meat and dairy (FAO, 2019). This high food import dependency makes them vulnerable to global food price rises, as happened in 2006–2008, with the economic recession that followed the financial crisis of 2008 further impacting food security in these vulnerable countries (FAO, 2016b; Mittal, 2009). By 2017, food imports in SIDS had reached US$5 billion dollars per year, a 50% increase in value since 2000 (FAO et al., 2017). While not all imported food is ultra-processed and calorie-dense such as processed meats, biscuits, ready meals like noodles and soft drinks, imported staples such as rice and fruit and vegetables are often more affordable and desirable than local produce (Connell et al., 2020). The agricultural industry of SIDS tends to be small scale, in particular at fresh food local markets where largely smallholder, high-cost producers with limited farmland compete against relatively cheap processed and fresh imports from industrial agricultural systems in countries like Australia and the USA (Connell et al., 2020). Small scale local food production results in limited investment, slow technological advancement, less diversification and insufficient economic viability in regional and global export markets, and therefore making SIDS' food systems highly vulnerable to natural disasters and economic downturns (FAO, 2016b). More recently, food security in SIDS has been and continues to be negatively impacted by the COVID-19 pandemic, as mitigation strategies reduced access to land, labour, tools and markets, and therefore reduced agricultural production and household dietary diversity despite some uptake in home gardening (FAO & ECLAC, 2020; Iese et al., 2021). This further exposed vulnerabilities related to reliance on food imports, impoverished local production, and for many countries an economic reliance on tourism (Hickey & Unwin, 2020).

Policy responses to this challenge within SIDS include the support of local food production to enable improved access and availability of less processed foods (IFPRI, 2015; UNSCN, 2016). The 2017 Global Action Programme on Food Security and Nutrition in SIDS (FAO et al., 2017), for example, aims to strengthen enabling environments for food security and nutrition; improve sustainability, resilience and nutrition-sensitivity of food systems; and empower people and communities for food security and nutrition. Examples of regional policies include the 2010 Caribbean Community (CARICOM) Regional Food and Nutrition Security Policy, which seeks to promote healthy Caribbean diets through increasing the production and availability of regionally produced foods, and the Regional Framework for Accelerating Action on Food Security and Nutrition in Pacific SIDS, which aims to strengthen the coherence and coordination of development partner support for food security and nutrition in Pacific SIDS (CARICOM Secretariat, 2010; Pacific Community, 2018).

While such commitments represent strong foundations for reaching food and nutrition security goals, they are cast broadly and generalised for all SIDS, and evidence from SIDS on key interventions that could support local food production initiatives is minimal, including evidence on the impact of local food production on population nutrition (Haynes et al., 2018). The state of local food production in each SIDS is unique and dependent on its own historical, social, political, cultural and geographical context (such as size and remoteness), and thus the effectiveness of commitments in each SIDS depends on such (Connell et al., 2020). For instance, a US study examining the facilitators and barriers of the development of local food markets found that the success of local markets depended on certain local conditions which are often not acknowledged during the development stages (Godette et al., 2015). These can include distribution systems, education and capacity for marketing of local food, uncertainty of regulations, and food safety requirements (Godette et al., 2015; Martinez et al., 2010). Likewise, Maples et al. explain that local food systems are influenced by characteristics of the population itself, including socioeconomic demographics, food ideology and degree of civic engagement (Maples et al., 2013).

In-depth understanding is needed of the political economic and socio-cultural factors that shape the specific contexts of local food systems and actors in SIDS to inform national policies on food and nutrition security (Barry et al., 2020; Singh-Peterson & Iranacolaivalu, 2018). To inform a wider project that aimed to develop methods to evaluate impact of local food production on local diets (Haynes et al., 2020), we investigated the contextual facilitators of and barriers to local community-based food production initiatives in two case settings: the Pacific Island country Fiji and St. Vincent and the Grenadines (SVG) in the Caribbean. Specifically, we sought to explore local food production networks and key stakeholders; the challenges and successes within and across each of the settings; and how barriers to success might be overcome.

## Materials and methods

### Settings and participants

We conducted in-depth, semi-structured interviews with key stakeholders involved in local food production and its supply chains in Fiji and SVG between August and November 2018. Fiji and SVG are both archipelagos of islands with similar economic standings and NCD burdens (Table 1).

**Table 1:** Selected demographic (CIA, 2020a, 2020b), economic (World Bank, 2019) and health (WHO, 2018) data on Fiji and SVG

	**Fiji**	**SVG**
**Region**	South Pacific	Caribbean
**Sub-region**	Melanesia	Lower Antilles
**Population**	883,480	110,210
**World Bank economic group**	Upper middle income	Upper middle income
**Major sectors**	Agriculture, tourism, sugar processing, textiles, copra, gold mining, lumber	Agriculture (starch), tourism, food processing, cement, furniture, textiles
**GDP per capita, PPP (current international $)**	11,004	12,307
**GDP proportion from agriculture (%)**	14	7
**Labour force in agriculture (%)**	44	26
**Raised blood glucose, adults aged 18 + (%)**	17	10
**Obesity** Adults aged 18 + (%) Adolescents aged 10–19 (%)	30 10	24 11
**Raised blood pressure, adults aged 18 + (%)**	20	23

Interviews in Fiji took place in its main island, Viti Levu. SVG interviews took place in St. Vincent (its main island) and Bequia (smaller island in the Grenadines close to St. Vincent). Populations of these two settings have diverse cultural backgrounds. In SVG, the population is mostly comprised of African descendants, with White, East Indian and Indigenous populations forming smaller proportions. The Indigenous Kalinagos or ‘Caribs’ tend to reside in the north of the island of St. Vincent; they are descendants of the island's original Indigenous population, which have reduced significantly in number during colonisation in the eighteenth century (Minority Rights Group International, 2007). In Fiji, the population is comprised mostly of Indigenous Fijians, also called iTaukei, who own about 89.75% of land in Fiji (iTaukei Land Trust Board, n.d.). Other major ethnicities in Fiji include Fijians of Indian and Chinese descent and Rotumans (Minority Rights Group International, 2017).

Stakeholders were purposively sampled from government, private and civil society sectors, including subsistence farmers and fishers (Table 2). Recruitment followed a process of cascading through the food chain, starting with local producers and tracing how and where their produce is transported, stored, processed, marketed and retailed. Individuals were identified through a combination of guidance from the local Ministries of Health, local contacts associated with the project, and through snowballing based on guidance from interviewees and speaking with people in the communities who worked in the local food chain.

**Table 2:** Participants by sector and level of food chain involvement in Fiji and SVG

	**Proportion of participants (%)**^**a**^	
	**Fiji (n = 32)**	**SVG (n = 20)**
**Sector**		
Civil society	1 (3)	3 (15)
Private^b^	26 (81)	18 (90)
Government	5 (16)	0 (0)
**Level within food chain**		
Production	26 (81)	9 (45)
Processing & transport	6 (19)	12 (60)
Marketing & retail	28 (88)	15 (75)

^a^ –Note that some participants overlapped in their sector representation and position in the food chain, so percentages do not necessarily sum to 100.

^b^ Including subsistence producers and smallholders

The roles of the participants included production (farming and fishing), processing (for example, producers of pepper sauce, coconut water, plantain/cassava/taro chips), transport, marketing, and retail (food shops and restaurants). Government officials were only available to be interviewed in Fiji. We also interviewed representatives of farming cooperatives, exportation companies and non-governmental organisations (NGOs) who support agriculture through funding, subsidising, transport and training. Throughout the manuscript, participant quotations are labelled as SVI# (for SVG) or FJI# (for Fiji).

### Data collection and analysis

The semi-structured interview guides (see Online Resource) focused on livelihoods through local food systems; factors that affect the operations of the local food system; keys and threats to the success of the local food system; environmental impacts of and on local food production; socioeconomic and health benefits of local food production; initiatives that support the local food system; and resources for and barriers to these initiatives. Our question and analysis are particularly relevant to three of the four pillars of food security (CFS, 2014), and contribution to them from local production. These are availability, accessibility and stability. Here, we considered less explicitly the fourth pillar, utilisation. However, in previous publications from the same project we have described aspects of utilisation in both settings (Guell et al., 2021; Haynes et al., 2020).

This was a collaborative research project between researchers from the Pacific Islands, the Caribbean region and the UK. All interviews were conducted by local researchers at interviewee farms, households and business establishments and lasted between 40 to 90 min. Interviews were audio-recorded and transcribed verbatim. Transcripts were analysed using a pragmatic approach of (a) initial deductive coding according to a predefined coding frame based on study objectives, and then (b) opened up for more inductive insights in reflexive thematic analysis (Braun & Clarke, 2019). Each country team at the University of the South Pacific and the University of the West Indies analysed their own transcripts as the main researchers in the first instance, identifying emerging relevant themes and salient quotations for illustration. Initial findings and unexpected insights were then discussed with the whole project team, including from the Universities of Cambridge and Exeter in the UK, in a group workshop. The analysis team used Dedoose software to allow for multi-person analysis across teams and countries (Dedoose Version 8.0.35, 2018).

### Terminology

The food systems in both settings largely included private home gardens producing food for own consumption (although also sharing, bartering and sometimes for market), smallholdings that provide for subsistence and livelihoods, and commercial farms (small in scale compared to import markets) (Haynes et al., 2020), typical for similar settings (Galhena et al., 2013). We describe formal networks as formalised economic or legal based relationships such as cooperatives, associations and business partnerships, and informal social networks as ad hoc, flexible and often opportunistic. We refer to processed and ultra-processed foods according to the NOVA classification foods that distinguishes unprocessed and minimally processed foods in the lowest category (at most, only fermented, pasteurised, boiled, dried etc.) to processed and ultra-processed meals, snacks and drinks partly or wholly produced using industrial techniques processes (Monteiro et al., 2016).

## Results

### Local food actors and interactions

As we set out to explore local food production networks and key stakeholders, a first step in the analysis was to trace local formal and informal networks of the food value chain. In this context, we define networks as relationships between multiple individuals and organisations that involve the exchange of information, infrastructure, finances or goods in relation to the production, supply and retail of foods. In both settings, smallholder farmers represent the largest producer numbers in the network for local food production, and serve a role in subsistence consumption, for sale within the local market, and regional and international export. Fresh produce, including main staples like yam, sweet potato, cassava, taro (Fiji) and dasheen (SVG), is grown in private backyard gardens for own consumption, on smallholdings that provide for subsistence and livelihoods, and on commercial farms. The local private sector of hotels, supermarkets and restaurants help to maintain demand for local fresh products, and farmers would try to diversify their produce, for example by growing tomatoes, cucumbers and lettuce, to meet demand, but imported produce would often be considered more varied, visually desirable or cheaper. Local food processing is a growing industry, although mostly small in scale, and includes *minimally* processed foods such as flours, milk, nuts, fresh fruit juices and culinary ingredients such as oils and dried herbs. Local *processed* food production includes salted nuts, sweetened juices, fried and salted chips, confectionaries (such as tamarind balls in SVG), pepper sauces, jams and chutneys. Depending on actual ingredients and methods of production, most of these would classify as ‘processed’ not ‘ultra-processed’ foods (Monteiro et al., 2016), but are often high in salt, sugar or fat content.

The Ministry of Agriculture is the main entity overseeing local food production in both countries, carrying out national programmes that include development and training, research, funding opportunities and veterinary services. Local and regional NGOs also play a major role in managing the supply and demand of products across production, processing and local and export consumer markets in both settings. Most organisations operate on a national level, with some regional (i.e. within the Caribbean) and international involvement with respect to funding and exportation.

In SVG, the Eastern Caribbean Trading Agriculture and Development Organisation assists small farmers in production planning and training of agricultural workers. Invest SVG, under the auspice of the Ministry of Finance, Economic Planning and Sustainable Development, plays a major role in assisting farmers in project planning and funding. There are many farming cooperatives in SVG which work together in supplying national and regional demands for local produce. Non-profit civil society organisations such as Richmond Vale Academy work with communities on poverty reduction, environmental conservation and climate change awareness, including creating organic demonstration farms and sustainable home gardens and contribute to national initiatives.

In Fiji, the Ministry of Agriculture offers a range of assistance to encourage production including consultations on proper farming procedures and soil assessments, subsidised tractor services, and provision and sharing of inputs such as new plant suckers. Agro Marketing Fiji Limited directly purchases root crops from rural farmers and assists in finding suitable markets for them in the hope of improving their standards of living. Local NGOs, most prominently the Foundation for Rural Integrated Enterprises and Development (FRIEND), play an important role by working directly with farming communities to understand their resources and products, and assist in incorporating them into the local value chain. They encourage traditional, Indigenous farming practices, including integrated cropping, and promote a backyard gardening programme.

### Local collaboration in a small market

A core function of formal and informal networks of local food producers, processors and vendors was to share resources and lessons. Such collaboration was facilitated through local governance structures, through civil society or informally across communities. In Fiji, sharing of resources, interdependence, reciprocity and collective community efforts are important Indigenous values and approaches. Stakeholders in both settings stressed that those working in local food production in both formal and informal networks must look at each other not as competitors but as partners. This necessitates understanding and appreciating everyone’s role in the food chain as important: *“we just have to work together […] so everybody gets something. Once we work together, it’s all good.”* (Restaurant manager, SVI17) Maintaining good relationships with partners by committing to requirements or requests was considered key to maintaining each cog of the food chain. Treating customers and workers with respect and *“work[ing] together with love”* (Farmer, FJI01) was considered important, not least as ill-treated staff were thought to work slower and produce less output.

In Fiji, stakeholders highlighted as a great facilitator of local food production the strong cultural value of ‘*Na Solesolevaki*’ underlying what was generally described as a strong sense of community and comradery between local food producers. *Na Solesolevaki*, where Indigenous farmers regularly come together to work on a particular farmer’s plantation as a group, was thought to bring joy in working as a team. They also believed this practice improved efficiency across farms, enabling them to share land and its produce, and share new and different ideas. The communal sharing of knowledge was commonplace and also a key cultural component of training workshops offered by the NGO FRIEND to local farmers and gardeners who could then return to their village to teach others. Fiji producers and processors generally described a positive relationship and a level of respect for one another. Farmers took advantage of initiatives provided by the agroprocessors and funded by government, which included providing steady, secure demand for crops such as cassava, agro-marketing for cassava products, and on a very practical level travelling to rural farmers and minimising their need for transport of produce to markets: *“We are going right to them [farmers] and buying from them. […] The government wanted us to go into them rather than them spending money coming over to the urban areas. They provide us the grant for us to go.”* (FJI20, Agroprocessor).

Government and key local food production partners in Fiji had also established local village committees consisting of village headmen and representatives of women, farmers, and fisherfolk who were trained on how to assess produce for their quality for health and organic status. They were seen as a key driver for the establishment of village community food production initiatives, and also operated as middlemen and quality control agents. Fiji stakeholders were generally pleased with the government-level commitment offered to them. Farmers were appreciative of their consultations on the use of manure and other farming practices*.*

Stakeholders’ experiences around sharing of knowledge and practices among actors and institutions within the local food production network differed between sectors and actors, but there was a particularly stark difference between Fiji and SVG. Although cooperation was highlighted as vital for strengthening the local food system, SVG stakeholders described a reluctance to work together. One participant explained that farmers often did not attend meetings of their farming groups, and when they do, “*they go home they’ll be like, ‘[sucks teeth] I don’t have time.’ [Laughs]. Or, ‘It’s too much work’”* (Vendor, SVI11) This reluctance to work together was connected to lack of trust and fear of competition between farmers.

*“There’s this mistrust and I’m not quite sure where it stems from. Because you always feel like you’re being, they always give you the impression that, you know, somebody’s going to cheat you.”* (Farmer, SVI16)

Stakeholders indeed lamented that buyers and sellers sometimes did not stick to agreements made, whether formal or informal, for example, if they found more attractive buying or selling rates elsewhere. As an example for this lacking loyalty, one farmer in SVG attempted to increase business through supplying local hotels with a group of other small farms to meet the high demand, as neither farm could meet the demand alone. However, the project failed as other farmers did not meet their commitments. The farmer’s conclusion was that *“They [other farmers] cut me out [by failing to provide sufficient quantities to meet the demand for the collective agreement]. So I said what I can’t produce [on my own], I just not going to sell.”* (Farmer, SVI16).

SVG stakeholders also reported more varied national-level and governmental support. Some reported low levels of trust and confidence in their government’s agricultural advice as being outdated and unsuited to the local terrain and crops (likely due to inadequate training), but appreciated business-oriented support provided through one government initiative and its willingness to address financial challenges of local farmers and agroprocessors. Invest SVG, under the auspices of the Ministry of Finance, Economic Planning and Sustainable Development, stood out for engaging and supporting farmers and food processors in providing guidance and advice on operating within the local food production network, and offering on-the-ground assistance for persons interested in or currently involved in agriculture through grants, hosting trade shows, and opening dialogue on marketing*,”and they come and help you.”* (Farmer and agroprocessor, SVI05).

The poorer cooperation in SVG was also explained by farmers lacking the initiative for open discussion and exchange. As one participant suggested, “*like me and you here sitting down and just having a conversation, talking about issues. People don't want to do that. So I have to, I have to blame the public as well.”* (Butchery manager, SVI09) This disconnect was also seen to result from the inability of people working in local food production to articulate their needs meaningfully.

“*The farmers lack the ability to articulate […] their situation. So a lot of the things that they need, they can’t speak for it. […] I am, I'm lucky to be able to articulate my position. But, but a lot of them not able to do that.”* (Farmer, SVI03).

In support for greater collaboration, the civil society organisation Richmond Vale Academy in SVG spearheaded the concept of ‘model farming’. These demonstration farms were set up in communities with the aim to provide experiential training, sharing, co-learning and adapting practices, between smallholders, and aim to ensure that existing and new farmers work efficiently, innovatively and with little environmental impact through peer learning. This small initiative resonated with local farmers. As one farmer explained: “*Education is, is critical! We need to have model farms […] And that’s what I’m trying to do myself. I’m trying to set up a model that would encourage young farmers [to emulate practices].”* (Farmer, SVI03).

### Seeking innovation in underinvested food systems

Cooperation with co-learning was seen as an important mechanism for innovation for many stakeholders, and innovation was seen as a key factor and aspiration towards strengthened local food production. Stakeholders urged farmers to update their practices to improve efficiency and mitigate environmental impacts from pests, diseases and erratic seasons causing flooding and draughts by using more greenhouses; by improving water management systems; by using green energy; and implementing topsoil conservation techniques. Indigenous co-farming through *Na Solesolevaki* and small-scale initiatives such as demonstration farms and training programmes were seen as facilitating the sharing knowledge and skills, formally and informally.

SVG stakeholders saw new technologies as particularly important for innovation. The owner of a processing company took pride in exemplifying sustainability through solar power energy by making people ask themselves, *“Hey can I do that with my business somehow?”* (Agroprocessor, SVI14) Likewise, a farmer felt rewarded when she found out that their organic farming techniques were being spread even inadvertently to their own workers:

*“So, actually [a local initiative] is trying to be a model of sustainability […] by showing that it’s possible to do it. I think it’s a way of teaching. […] By hearing us talking about how you should grow organic all the time, […] they just tell us, like, ‘Yeah, so now I stopped using pesticides,’ or ‘I stopped using the weed killer.’”* (Teacher/farmer, SVI20)

Stakeholders also recommended moving beyond the current print and word-of-mouth communication channels, and including social media as a platform for improving connections between different strata in the local food production industry, helping to bring people together on local events, initiatives and opportunities. They also suggested that innovation could be stimulated through finding new ways to appeal to youth to encourage them to get involved in agriculture, and in return reap benefits from their affinity to new technologies to modernising farming practices.

*“You have to, kind of, try and speak their language […] There’s a lot of technology involved and that’s what the young people like. They have Apps now […] We can’t just say, oh, that grass look a little yellow, it going need some manure.”* (Farmer, SVI16)

In Fiji, rather than focusing on new technologies for innovation, stakeholders suggested that a young, climate change aware generation could be attracted by highlighting the need for more sustainable and ‘traditional’ farming practices without the heavy applications of fertilisers, herbicides, pesticides and other harmful chemicals. Stakeholders saw a specific role for government in this, recommending that local food production using alternative, regenerative or organic practices instead of orthodox ‘industrial’ processes of farming should be integrated more in the national school curricula to attract a new generation of food producers. Stakeholders also called for more capital investment to develop a more climate resilient food system to address challenges such as flooding and soil erosion form extreme weather events. Likewise, Fiji stakeholders believed that government had a role in supporting sustainable and organic farming through awards and incentives and this would improve the quality of their produce. Instead of observing progress towards this aim, Fijian stakeholders worried about a strong push from agencies, funding bodies and therefore communities towards mono-cropping of imported species of plants like potatoes, peppers (capsicums) and broccoli instead of mixed cropping of local food species such as yams, wild ferns and nuts. One stakeholder explained that “*we are not sustaining enough of our own varieties”* and *“despite the aid, we don’t have corporates looking into the potential of Fiji crops.”* (Processor and exporter, FJI09) Local bodies and stakeholders *“don’t consider traditional agriculture as agriculture”* (Processor and exporter, FJI09). Instead, local NGOs follow prescription from international aid and fail to emphasise the range of staple Fijian root crops or leafy vegetables.

Stakeholders in Fiji and SVG felt that a key barrier to strengthening the local food system was insufficient technical knowledge in state institutions to support economically viable and sustainable local food production, and that this has led to inadequate practices among farmers. Stakeholders pointed to such inefficient and outdated practices such as the extensive use of fertilisers and planting techniques that are sometimes poorly suited for the terrain, leading to lower yield, higher costs, and heavily impacting soil quality and erosion, and identified a number of issues in relation to the role of agricultural extension officers. There were few of them, there was a lack of trust in extension workers to adequately train farmers, and those who trained farmers focused on techniques that they had learnt overseas but were often not appropriate to the local geography and other contextual factors shaping small scale farming. Examples of such challenges requiring technical expertise not necessarily acquired abroad included bush fires in rural areas of Fiji and uncontrolled slash and burn farming in SVG, that would require soil management and other mitigation strategies.

Stakeholders therefore suggested that farmers were ill-equipped with the right skills, but stakeholders were also worried that farmers were not interested in or could not contribute to sustainable farming practices mitigating long-term harm to land and agriculture because of land tenure challenges. Many farmers leased their farmland, so their focus was on short-term profitability instead of long-term soil quality and longevity. One SVG farmer explains that “*a lot of people do not care [about the] land because they do not own land. […] The largest threat to the farmers themselves are farmers. Because they are the ones that are really destroying the surface.”* (Farmer, SVI03). In Fiji, one stakeholder highlighted the precarious situation of tenant farmers:

*“The land that I’m staying in is not considered to be under a formal village setting. When residential developments started to occur, I had to stop planting at the piece of land that I was using. So currently I’ve been buying food from the market”.* (Farmer and fisherman, FJI32)

Finally, stakeholders in both settings connected vulnerabilities in local food production to their weak national economies that hindered concerted investment. This included low incomes resulting in emigration of skilled workers, and fluctuating availability of supplies, such as animal feed, making animal husbandry haphazard. One stakeholder explained that the Fijian *“agriculture department don’t even have enough seeds or storage.”* (Processor and exporter, FJI09) Fijian villagers who travelled to the market to vend must travel by bus without adequate storage of their produce, leading to damaged produce. Lacking financial investment in good quality infrastructure to support local food production systems – from roads to shipping and airfreight between islands – was considered to be particularly problematic in both settings and seen to restrict profitability and lead to food wastage.

***“****[In other countries] it is unthinkable that a farm doesn’t have a drivable road! It’s unthinkable they don’t have road or water and electricity. But these are the same farmers we’re compare, competing with. As a matter of fact, they have subsidized fuel. […] The agricultural policy in Europe, there’re heavy subsidies going into farms!”* (Farmer, SVI03)

*“They don’t have farm roads. […] Those are the kind of infrastructure that should be there that will help us reach out to the remote islands. […] We don’t go right to the farm. We don’t send our guys right to the farm even if it’s a kilometre away. That’s a barrier to us eh.”* (Agromarketer, FJI20)

The latter quote showed that even initiatives like the earlier mentioned scheme of Agro Marketing Fiji Limited to directly buy from producers to overcome transport challenges only made small inroads in addressing these structural barriers. Similarly, agroprocessors of coconut oils, chips and sauces complained of the high costs of processing, particularly the machinery required, which limits the expansion of their businesses. One SVG farmer claimed that instead of consuming imported processed foods, *“we could process our own thing here and make it as tasty but, again, is the money to do it, you understand me?”* (SVI05). A Fiji stakeholder interested in food processing also explained *“I have been to China a couple of times to see some big machines […] but it is expensive”* (Exporter, FJI37), and has been unable to purchase any and upgrade his system. Stakeholders in SVG called for *“concessions, fiscal incentives, you know, ten years, fifteen years, fiscal incentives”* (Agroprocessor, SVI07) that are commonplace in higher income countries. While a drive for innovation and marketing local products was clearly evident, these financial barriers seemed difficult to overcome.

### Capitalising on local pride

Despite the underfunded position of local food production, stakeholders felt that local foods were generally appreciated and considered key to the resilience of the local economies in both settings. Because Fiji and SVG are traditionally agricultural societies, stakeholders suggested that while the local population might well consume a high percentage of imported products, they nonetheless preferred local foods as an act of patriotism and loyalty to their people. Stakeholders also praised locally produced fresh produce as healthier than imported produce on account of their assumed lower content of chemicals – be it fertilisers, pesticides, preservatives, or appearance enhancers. In both settings, stakeholders appreciated that consumers might not be aware of ubiquitous use of chemicals in local food production, yet they also attributed superior taste and viability of their local produce, for example connected to their rich, volcanic soil in SVG. One stakeholder stated that *“You’ll, you forget a, a stick on the ground, you come back it’s growing.”* (Farmer, SVI03) Similar sentiments were reported in Fiji. An owner of a large ginger farm believed that the healthy non-polluted environment is an element of success of his ginger demand: *“One of the main reasons why our customers like our Fiji-grown ginger is because Fiji enjoys a better reputation with the environment, and with our great team and environment here, no pollution.”* (Food exporter, FJI38) Healthiness was also connected to beliefs around nutritiousness, claiming that local foods score *“really high on nutrition charts*” (Processor and exporter, FJI09) with examples like *rourou* (taro leaf stew) suggested to prevent anaemia in Fiji. In our companion piece to this study, we also found ambiguous consumer preferences in both settings, of highly valuing local foods “and yet increasingly consuming shop-bought, processed and imported foods […] making trade-offs between health and convenience, and navigating uncertainties over the risks and benefits of different food types and sources”(Guell et al., 2021, p.7).

Stakeholders were keen to emphasise the importance of understanding and harnessing the high social value placed on local produce—and its connection to land, people and country—as a symbol of national pride and therefore an important strategy towards addressing the increasing consumption of imports and strengthening local food production and processing. A marketing agent explains *“they trust it, it's their country, they trust their soil more than what may be coming in. […] It's a, more of a sign of patriotism.”* (SVI02) Even processed foods such as pepper sauces, chips, oils and jams that are made from locally produced ingredients were described as preferred over imported ones. Stakeholders felt that this support of *“our own people”* (Agroprocessor, SVI07) is a key element of success for local food production. Promotion of local Fijian products is made through the use of conspicuous labels denoting that it is made in Fiji, and a major SVG supermarket has regular campaigns preferentially promoting local products.

Although highlighting these benefits for and preferences of local populations, stakeholders suggested that commercialisation and ultimately export of locally produced food were paramount for success. Local producers hoped for their products to be marketable enough to be sold out of country, noting that a “*sustained profitable market”* (NGO coordinator, SVI01) is a first step for a successful local food production industry*.* It is thought that available and sustained markets for local food production will encourage people to get involved in local food production and for those already involved, to improve product quality. Stakeholders in SVG called for their Ministry of Tourism to engage in local food production by marketing this unique industry to visitors. In Fiji, the continued empowerment of farmers to commit to shifting from small-scale subsistence to commercial production to increase economic rewards was considered critical:

*“So, I look at that transition as a very important transition, you know, it’s the transition of a mind, ah instead of looking at production purely from a subsistence perspective, now they need to go one up, you know, ah for a more commercial perspective.”* (Ministry of Agriculture, FJI13)

Despite this pride in the product and eagerness for capitalisation, stakeholders worried that local food *production* as a livelihood was less favourably regarded and increasingly seen as excessively hard work with little payoff. As one stakeholder put it, *“it’s no longer sexy to farm,”* (Farmer, SVI03) and youth tended to view farmers unappealingly “*as somebody with water boots and a hoe in their hand, a crooked back.”* (Farmer, SVI16) Instead, farming was seen as a transitional job, a *“stepping stone”* (Farmer, SVI16) to a job or career of greater significance or easier work. Attracted by professional careers, stakeholders feared young people *“rather go and adjust their collar and tie and go to work at the bank, or work behind somebody desk.”* (Restaurant manager, SVI19) Similarly, stakeholders appreciated that while consumers might voice preference for local fresh produce as healthier than imports, the economic reality was that their actual choice was limited when considering availability and affordability of certain products, in particular fresh produce that was often more expensive. A restaurant manager cautioned that even tourists were reluctant to buy from local vendors because of their high prices: *“Yes, they [tourists] excited, you know, they see local produce, it’s locally grown and thing, but then the prices are ridiculous. They feel like they’re being robbed.”* (Restaurant manager, SVI08).

## Discussion

### Summary of findings

This study aimed to investigate the lessons that can be learned for strengthening local food production in Fiji and SVG from the perspective of local food system actors. Imports of ultra-processed foods are significant drivers of malnutrition and dietary change, and imports of fresh produce outcompete local fresh food production and further exacerbate ill-health due to poorly accessibly and unaffordable locally produced fruit and vegetables (FAO, 2019). Our stakeholders’ perspectives described a local food system operating within formal and informal networks of cooperation to share resources and aim for innovative practices. However, stakeholders described how competition within these networks, weak national economies, insufficient technical knowledge, and lacking political will and selective and slow financial commitments have hampered investment opportunities and infrastructure development in local food production. While local produce was valued and marketed as a symbol of national pride, increasing preference for imported foods, depreciating attitudes towards farming as a way of life and little knowledge, technical and financial support for growing local crops and sustainable farming practices were cause for concern.

### Harnessing governance to strengthen local food systems

Many of the barriers highlighted by stakeholders in SVG and Fiji have been previously highlighted (Connell et al., 2020; Guariguata et al., 2020; Saint Ville et al., 2015). SIDS’ geographical isolation, limited land space, weak economies, and slow technological advancement in local food production undermine efforts to gain a foothold in export markets and compete against imported foods (FAO, 2014). The concern for sustainability of local food production in SIDS is widely acknowledged and the Food and Agricultural Organization is actively involved in supporting their food security and agriculture (FAO, 2014). SIDS global vulnerabilities such as to impacts of climate change are often the focal theme in these discussions and efforts (FAO, 2016a), and global policy efforts of the World Trade Organization’s Uruguay Round (WTO, 2019b) and later Doha Round (Tu'akoi et al., 2018; WTO, 2019a) aimed to reduce trade-distorting agricultural subsidies and tariffs that placed developing countries at a disadvantage. Yet it is important to consider the local governance forces at play which can counter these efforts.

Although there is substantial effort and investment of civil society actors and private enterprise to make local food systems accessible and resilient, the role of the state seems crucial. Stakeholders in our study were particularly concerned about the significant policy marginalisation of the domestic agricultural sector, with little funding, including investment in up-to-date knowledge of sustainable and climate change resilient agriculture and willingness to abandon outdated technologies and processes such as monocropping of imported species and the use of fertiliser, herbicides and pesticides. As Sonnino (2013, p.4) suggests, it is the “presence or absence of governance arrangements that can sustain local food networks over space and time”, including its role “in constructing and supporting markets for local food products”. While global trade and corporate power works against such efforts and marginalises or even displaces local produce, government action can still act against such forces and dynamics. As our stakeholders suggested, this may be through investment in infrastructure, subsidisation, taxation or developing knowledge and skills within government for innovative local production. Barriers to this are described in the literature across SIDS settings, including little political appetite for regulation and market intervention at the backdrop of neo-liberal economic models (Connell, 2020). At the same time, non-state actors such as producers, processors and civil society may be marginalised, in particular in terms agenda or priority setting processes that could counter such politics and would foster inclusive and socially just agri-food governance (Siegel & Bastos Lima, 2020). Moreover, an underfunded or unsupported food system creates further barriers for non-state actors for knowledge exchange and cooperation for resource mobilisation or greater participation (Saint Ville et al., 2017).

Concerted state support is also increasingly required to address climate change adaptation. Stakeholders mentioned inadequate investment in water management systems, and water insecurity is an important challenge in rain-fed smallholder farming systems in SIDS (FAO, 2019). It is likely in the future, with climate change, that both extreme rainfall events and periods of drought, will increase in importance. Local governance mechanisms such as traditional governance systems in Fiji need to be considered as vital levers in strengthening local food systems and sharing knowledge about climate resilient food production practices. In Fiji, most the lands and fishing areas (*qoliqoli*) are owned by iTaukei through the customary ownership in Fiji (iTaukei Land Trust Board, n.d.), and the Indigenous *Na Solesolevaki* practice of working together and sharing resources (including land with landless) and experience and expertise might at least fill some of the more formal national governance gaps. Such culturally and communally embedded governance structures seemed missing in SVG and thus unable to compensate for the lack of state attention.

### Harnessing social values to strengthen local food systems

Stakeholders across our study sites identified meaningful cooperation between food systems actors as an important mechanism towards innovation and resilience, including through self-started farming cooperatives, demonstration farms in SVG and the spirit of working together *Na Solesolevaki* in Fiji (which has proven vital during the COVID-19 pandemic (Iese et al., 2021))*.* However, stakeholders also shared experiences of fractured relationships, distrust and conflict between different food producers, processors and vendors, particularly in SVG, and as also reported in other Caribbean settings (Lowitt et al., 2015a, 2015b; Saint Ville et al., 2017). Lacking trust might need to be at least partially understood in the historical context of indentured labour and slave agro-economies that underlie the emphasis on self-sufficiency and individualism in Caribbean smallholder farmers (Lowitt et al., 2015a, 2015b). Contemporary political economic factors that may explain distrust include that formal and informal networks of diverse stakeholders with differing interests and access to resources operate in a highly open and underfunded market. As Saint Ville and colleagues (2017) found in their exploration of stakeholder engagement within the agri-food system in neighbouring Saint Lucia, collaboration processes were often top down and lacking representation of smallholder farmers, and therefore fostering distrust, and would require explicit structures to govern greater co-learning and collective action. Similarly, Lowitt and colleagues (2015a, p.1367) found “a systemic lack of access to finance, markets, and knowledge networks” that was compounded by “a pervasive lack of trust reported between actors and institutions throughout the agricultural innovation system […] in the Caribbean.” They suggest that enabling ‘communities of practice’ with shared values, needs and priorities could provide such structures to foster social and collaborative relationships and collective action in smallholder famers (Lowitt et al., 2015a, 2015b).

One shared social value stakeholders in *both* settings highlighted was an appreciation of ‘local’ produce by local populations, speaking to a literature that highlights that local food produce captures a range of values from environmental sustainability to social embeddedness and healthy nutrition (Sonnino, 2013). This pride was less seen in appreciation of the agricultural sector itself, and its important role for local nutrition and health, despite some regional and international policy efforts (FAO, 2019), and there seems to be a need to extend national ‘pride in local produce’ campaigns to government ministries, agri-food organisations and funding agencies in supporting local species and sustainable growing practices rather than placing the onus solely on the consumer.

In tourism-dependent SIDS, a further value that could be harnessed could be around presenting ‘local food culture’ to tourists. However, perhaps more important here is the monetary value of local produce for local hotels and restaurants as well as for export markets. Pride in ‘local’ then has a commercial benefit to local producers to strengthen their business viability in a marginalised market. Agro-tourism is a well-regarded industry that can synergise the agricultural and tourism markets in countries like Fiji and SVG, and ultimately lead to increased development of both industries while also increasing foreign reserve (FAO, 2012). In SVG, local value chains of coconut water, cassava and sweet potato have already experienced increased market demand and such local agricultural diversification is supported by the 2016–2019 FAO Country Programming Framework (FAO, 2018). However, production for a tourist market may render such produce unaffordable for local populations when willingness and capacity to pay is much higher by tourist businesses. It may also divert high quality fresh produce away from local consumption, particularly when production volumes are small, plant scheduling systems are ad-hoc and there is low information exchange to enhance coordination between relevant actors. Production for the tourist market then has little benefit to the food security and nutrition of local populations. Stakeholders also warned that tourism encourages the production of produce of little environmental value to their countries, as hotels and restaurants also would like resource-intense produce such as lettuce or broccoli on their menus and undermining their own efforts to develop resilience in their food systems. Nonetheless, ‘going local’ can also be a political endeavour of small scale producers and organisation against corporate practices and large scale production, and alternative models such as farm-to-table establishments could emphasise sustainable practices and the importance of relationships with local consumers (Beingessner & Fletcher, 2020). In Fiji, the NGO FRIEND runs a commercially successful restaurant with healthy local dishes cooked in traditional ways that is very popular with the local population. Stakeholders’ appeal to engage more with young people and harness creativity and innovation in a new generation also points towards opportunities in this direction.

## Conclusion

There are multiple social, economic and political factors that challenge local food production in Fiji and SVG, from limited investment in weak economies to mistrust of actors in the local food production network and dominant import markets. Community and civil society efforts such as demonstration farms, Indigenous shared farming practices and governance structures, and informal networking between local food producers and processors might make small inroads in strengthening local food systems. However, this needs to be considered against the backdrop of agri-food systems that use predominantly top–down approaches, with minimal participation from consumers, farmers and NGOs, and a growing dominance of food imports and supermarkets (Lowitt et al., 2016). The COVID-19 pandemic has, of course, underscored the precarious nature of food security in many SIDS and the desirability of greater self-sufficiency and local food security and local food markets even further (FAO & ECLAC, 2020; Hickey & Unwin, 2020). While diverse stakeholders work towards strengthening local food production in SIDS, strong governance structures are needed that prioritise local produce over corporate and import markets, assist collaboration and co-learning, and support alternative approaches to agro-food practices.

## Supplementary Information

Below is the link to the electronic supplementary material.

Supplementary file1 (DOCX 26 KB)

## Data Availability

This is qualitative data collected in small settings, and is therefore not fully anonymous. The corresponding author can be contacted to discuss limited access to the transcripts.
