# Supplementary material for: Perspectives on strengthening local food systems in Small Island Developing States
Source: Food Secur. 2022 May 4;14(5):1227–40. doi: 10.1007/s12571-022-01281-0 (PMC9067893; doi:10.1007/s12571-022-01281-0)
Supplement: Supplementary file 1 — Supplementary file1 (DOCX 26 KB) [file 12571_2022_1281_MOESM1_ESM.docx]

**ONLINE RESOURCE: Interview guides**

**FOOD SECURITY**

**Harnessing cooperation and localism for innovation to overcome underfunded and marginalised local food systems: lessons learned from local food actors in Small Island Developing States**

Cornelia Guell^1^ (corresponding author; c.guell@exeter.ac.uk), Catherine R Brown^1^, Otto W Navunicagi^3^, Viliamu Iese^3^, Neela Badrie^4^, Morgan Wairiu^3^, Arlette Saint Ville^5^, Nigel Unwin^1,6^ on behalf of the Community Food and Health (CFaH) team^7^

1 - University of Exeter, European Centre for Environment & Human Health, Truro, United Kingdom

2 – The University of the West Indies, George Alleyne Chronic Disease Research Centre, Bridgetown, Barbados

3 – The University of the South Pacific, Pacific Centre for Environment and Sustainable Development, Suva, Fiji

4 – The University of the West Indies, Department of Food Production, St. Augustine, Trinidad and Tobago

5 - The University of the West Indies, Department of Geography, St. Augustine, Trinidad and Tobago

6 – University of Cambridge, Medical Research Council Epidemiology Unit, Cambridge, United Kingdom

7 – See acknowledgements section

**Interview guide for general stakeholders**

| **Main Questions** | **Follow-up/prompts** |
| --- | --- |
| *Note: generic interview guide to be adapted for each stakeholder as appropriate* | |
| In what way or ways are you involved in producing or supplying food to people in St Vincent / Fiji? | What is your role? - along the supply chain, which sector, for how long have you been involved  *If they are not actual producers / fishers*: how much of the food that you process / supply / retail is grown / produced / caught locally? |
| We are particularly interested in locally produced food in your setting.  What kind of food is locally produced? | Have there been any changes on locally produced food over time? |
| How do people make a living in local communities through local food production? | Production (including growing, fishing, rearing)  Processing  Transport  Financing/marketing  Trade/vendors  Have there been changes to those involved in the supply chain over time? |
| *Tailored question to stakeholder role, e.g. any of these:*  What are important factors influencing agriculture, fishing, storage, transport, food marketing, retail, food prices? | What is most important? What is less important? Why? |
| What are key elements of success to strengthen the local food supply? | Particular organization, players, community |
| Who are the main organizations, sectors, stakeholders, partners working together? | *Prompts if no answers forthcoming:* governmental, civil society, private |
| What are common threats to the local food supply? | *Prompts if no answers forthcoming:* Globalization, international trade, aid; social/population change |
| What are common threats to businesses/individuals involved in:  *(disaggregate as appropriate to each stakeholder)*    - food production?  - food processing?  - food storage?  - food transport and distribution?  - food retail and food service? |  |
| How are these threats addressed in your setting? | Who addresses these threats? |
| Can you particularly comment on environmental impacts *on* local food production? | How does climate and environmental impacts/ changes affect your role in food system? |
| What is the environmental impact *of* local food production? | Can you tell us examples?  *Prompts if no answers forthcoming:* Soil, water; erosion/pollution, pesticides/fertilisers; carbon emission; deforestation  For whom? |
| What do you think are the economic and social benefits of local food production for those involved in the process? | Can you tell us examples? Benefits for whom (producers, vendors, consumers, yourself)? |
| Do you think that there are any health benefits of the local food system for communities? | Can you tell us examples?  *Prompts if no answers forthcoming:* Food security – adequate nutrition; link to NCDs/obesity? |
| Can you tell us about particular *initiatives* you know of, or have been involved with, that support the local food system? | What are they typically doing? (e.g. subsidies for subsistence fishing/farming; providing equipment etc.)  What is/was their aim? (e.g. improve food security, economic livelihoods, health)  How successful would you say these initiatives are in achieving their aims?  What kind of initiatives – types, aims – are most common and relevant in your setting?  Who initiated, ran, financed these? (civil society, government, private, local, regional, international)  How did they come about, how was it implemented, what was/is it like running it, what is its future/legacy |
| What are the resources and support available for such initiatives? | *Prompts if no answers forthcoming:*  Local, communities, government, international aid/NGOs (not just financial)  What makes them sustainable? |
| What are the common barriers of these initiatives? | *Prompts if no answers forthcoming:*  Social, economic, environmental  Planning, implementation, acceptability, sustainability, resources  *If example of unsuccessful initiative:* What led to their breakdown? |
| Thank you very much. These are our questions.  Is there anything else you would like to add you think we forgot to discuss today?  Which other individuals (and their organizations) should we be aware of / interview as a key informant? | Why would you recommend this person? |
| Thank you! | |

**Interview guide for representatives of specific initiatives**

| **Main Questions** | **Follow-up/prompts** |
| --- | --- |
| *Note: generic interview guide to be adapted for each stakeholder as appropriate* | |
| Can you tell us about *initiative x*? | Can you describe the initiative?  What its aim? (e.g. improve food security, economic livelihoods, health) |
| How did it come about? | Who initiated, financed the initiative? (e.g. civil society, government, private, local, regional, international)  How was it implemented? |
| Who runs the initiative? What actors are involved? What is your role in it? | What is it like being involved in it? What is its future/legacy? |
| What are the resources and support available for your initiative?  *For interviewers: for local farmers/fishers, this could be rephrased:* what support do you get by being part of this initiatives? Who helps you? | *Prompts if no answers forthcoming:*  Local, communities, government, international aid/NGOs (not just financial)  What makes them sustainable? |
| What are other important factors influencing your initiative?  What are key elements of success? | Could be any activities, actors and relationships along food value chains leading from production via processing, packaging, storage, transport, to retail, service and consumption.  Local skills, access to land/water, climate/environment |
| What are the common barriers of your initiative? | *Prompts if no answers forthcoming:*  Social, economic, environmental  Planning, implementation, acceptability, sustainability, resources  Threats to production, processing, storage, distribution/transport, marketing, food retail/services, consumption |
| We are particularly interested in the impacts initiatives like yours might have on local communities. |  |
| What do you think are the economic and social benefits of your initiative for those involved in the process? | Can you tell us examples?  Benefits for whom (producers, vendors, consumers, yourself if applicable)? |
| Do you think that there are any health benefits of your initiative for communities? | We are particularly interested in food security in relation to adequate nutrition; link to NCDs/obesity |
| Do you think your initiative might have an environmental impact? | Positive: e.g. improve soil fertility  Negative: e.g. soil, water; erosion/pollution, pesticides/fertilisers; carbon emission  For whom? |
| And in turn, do you think environmental factors impact *on* your initiatives? | Climate change, extreme weather events |
| Can you tell us about *other initiatives* you know of, that have been involved with that support the local food system? | What are they typically doing? (e.g. subsidies for subsistence fishing/farming; providing equipment etc.)  What is/was their aim? (e.g. improve food security, economic livelihoods, health)  What kind of initiatives – types, aims – are most common and relevant in your setting?  Who initiated, ran, financed these? (civil society, government, private, local, regional, international)  How did they come about, how was it implemented, what was/is it like running it, what is its future/legacy |
| How does your initiative compare to other initiatives in your setting if there are any? | Similar success/barriers/support? |
| Thank you very much. These are our questions.  Is there anything else you would like to add you think we forgot to discuss today?  Which other individuals involved in your initiative that we should interview as a key informant? | Why would you recommend this person? |
| Thank you! | |
